# Polypeptide Globular Adiponectin Ameliorates Hypoxia/Reoxygenation-Induced Cardiomyocyte Injury by Inhibiting Both Apoptosis and Necroptosis

**DOI:** 10.1155/2021/1815098

**Published:** 2021-07-08

**Authors:** Kaiyi Zhu, Jia Guo, Xiaoxue Yu, Que Wang, Chao Yan, Quan Qiu, Weiqing Tang, Xiuqing Huang, Hongna Mu, Lin Dou, Yunfei Bian, Qinghua Han, Tao Shen, Jian Li, Chuanshi Xiao

**Affiliations:** ^1^Center for Hypertension Care, Shanxi Medical University First Hospital, Taiyuan, Shanxi 030001, China; ^2^Shanxi Medical University, Xinjiannanlu 56, Taiyuan, Shanxi 030001, China; ^3^The Key Laboratory of Geriatrics, Beijing Institute of Geriatrics, Beijing Hospital, National Center of Gerontology, National Health Commission, Institute of Geriatric Medicine, Chinese Academy of Medical Sciences, Beijing 100730, China; ^4^Department of Cardiology, The Second Hospital of Shanxi Medical University, 382 Wuyi Road, Taiyuan, China; ^5^Key Laboratory of Cardiovascular Medicine and Clinical Pharmacology of Shanxi Province, Taiyuan 030001, China

## Abstract

Adiponectin is a small peptide secreted and a key component of the endocrine system and immune system. Although globular adiponectin protects myocardial ischemia/reperfusion-induced cardiomyocyte injury, the protective mechanisms remain largely unresolved. Using a neonatal rat ventricular myocyte hypoxia/reoxygenation model, we investigated the role of its potential mechanisms of necroptosis in globular adiponectin-mediated protection in hypoxia/reoxygenation-induced cardiomyocyte injury as compared to apoptosis. We found that globular adiponectin treatment attenuated cardiomyocyte injury as indicated by increased cell viability and reduced lactate dehydrogenase release following hypoxia/reoxygenation. Immunofluorescence staining and Western blotting demonstrated that both necroptosis and apoptosis were triggered by hypoxia/reoxygenation and diminished by globular adiponectin. Necrostatin-1 (RIP1-specific inhibitor) and Z-VAD-FMK (pan-caspase inhibitor) only mimicked the inhibition of necroptosis and apoptosis, respectively, by globular adiponectin in hypoxia/reoxygenation-treated cardiomyocytes. Globular adiponectin attenuated reactive oxygen species production, oxidative damage, and p38MAPK and NF-*κ*B signaling, all important for necroptosis and apoptosis. Collectively, our study suggests that globular adiponectin inhibits hypoxia/reoxygenation-induced necroptosis and apoptosis in cardiomyocytes probably by reducing oxidative stress and interrupting p38MAPK signaling.

## 1. Introduction

Acute myocardial infarction is a worldwide public health problem [1, 2]. Currently, reperfusion therapies remain effective treatments for acute myocardial infarction. Myocardial ischemia/reperfusion also results in largely unavoidable injury in cardiomyocytes [3, 4]. Major causes for myocardial ischemia/reperfusion injury include calcium overload, excessive reactive oxygen species (ROS) production, and inflammatory factor release, all together ultimately leading to cardiomyocyte death and consequently myocardial dysfunction [5–9]. Apoptosis and necroptosis have been thought to be crucial in myocardial ischemia/reperfusion injury by regulating various intracellular signaling pathways [10]. Molecular mechanisms responsible for myocardial ischemia/reperfusion injury are still unclear. The understanding of these regulatory mechanisms may unveil novel therapeutic approaches for managing myocardial ischemia/reperfusion injury.

Apoptosis, also known as type I programmed cell death, occurs in myocardial ischemia/reperfusion injury via either the death receptor-mediated extrinsic pathway and/or the mitochondria-mediated intrinsic pathway [11]. On the other hand, necroptosis is a form of regulated cell death that is initiated by perturbating extra-, or intra-, cellular homeostasis in conjunction with the activation of MLKL, RIP3, and RIP1 [5, 11]. Many specific death receptors, such as FAS, TNFR1, or pathogen-recognition receptors, are activated, resulting in inflammation and cell death [12]. Necroptosis is also involved in many serious cardiac pathological conditions, including myocardial infarction, ischemia-reperfusion injury, and heart failure [13].

Adiponectin is a small peptide secreted by adipocytes and abundantly present in plasma, which is defined as a key component not only of the endocrine system but also of the immune system. Adiponectin exists as either a full-length 30 kDa protein circulating in trimeric, hexametric, and higher-order complexes or a fragment containing the globular domain of adiponectin (gAd) that exhibits metabolic activity in a pathophysiological setting [14, 15]. Major biological activities of gAd in lipid and glucose metabolisms include potent insulin-sensitizing, antiatherogenic, and anti-ischemic injury and antiapoptotic properties [16]. We previously reported that gAd attenuated vascular calcification by inhibiting endoplasmic reticulum stress. Additionally, we have also shown that gAd alleviated hypoxia-reoxygenation-induced injury in cardiomyocytes in vitro and in vivo by upregulating endoplasmic reticulum Ca^2+^-ATPase activity and inhibiting endoplasmic reticulum stress [17–19]. Evidence available for how gAd protects against cardiomyocyte necroptosis induced by myocardial ischemia/reperfusion injury is extremely limited.

In this study, we comparatively investigated necroptosis and apoptosis in neonatal rat cardiomyocytes and the protection and potential molecular mechanisms of gAd following hypoxia/reoxygenation *in vitro*.

## 2. Materials and Methods

### 2.1. Reagents

Dulbecco's modified Eagle's medium (DMEM) and fetal bovine serum (FBS) were obtained from Thermo Scientific, Waltham, MA, USA. Hank's balanced salt solution (HBSS), rat recombinant globular adiponectin (gAd), Z-VAD-FMK, necrostatin-1, dihydroethidium, and 3-(4,5-dimethylthiazol-2-yl)-2,5-diphenyltetrazolium bromide kit were purchased from Sigma-Aldrich, St. Louis, MO, USA. Antibodies against cleaved caspase-3, caspase-3, RIP1, RIP3, NF-*κ*B, p38MAPK, phosphorylated-NF-*κ*B, phosphorylated-p38MAPK, Bcl-2, Bax, and GAPDH as well as HRP-conjugated anti-rabbit IgG antibody were obtained from Cell Signaling Technology, Inc., Danvers, MA, USA.

### 2.2. Rat Primary Cardiomyocyte Culture

Female pregnant Sprague-Dawley rats were obtained from the SBF Biotechnology Co. Ltd., Beijing, China. The use of animals for this study was approved by the Ethical Committee of Shanxi Medical University in accordance with the internationally accepted principles of laboratory animal use and care. Neonatal rat ventricle cardiomyocytes (NRVMs) were isolated from rats 1-3 days after birth and cultured as previously described [20]. In brief, NRVMs were plated and selected in DMEM supplemented with 10% FBS with 0.1 mM 5-bromo-2-deoxyuridine. To study the effect of gAd, cardiomyocytes were incubated with vehicle (PBS) alone (control group) or 2 *μ*g/ml gAd under normoxic condition; cardiomyocytes were treated with hypoxia/reoxygenation (see below) and cultured with vehicle alone or 2 *μ*g/ml gAd, which was added into the wells for 24 h followed by hypoxia/reoxygenation treatment. PBS containing 0.5% BSA was used as the vehicle control.

### 2.3. Hypoxia/Reoxygenation Culture

Culture medium for NRVMs was replaced with high glucose DMEM with 1% FBS when the cells reached a confluence of 80-90% and were cultured for approximately 24 h prior to hypoxia exposure. Then, culture medium was replaced with low glucose DMEM, and the cells were cultured in a trigas incubator (HERAcell VIOS 160i, Thermo, USA) saturated with 5% CO_2_ and 1% O_2_ at 37°C for the indicated time periods followed by exposure to 5% CO_2_ and 95% air (*v*/*v*) at 37°C for reoxygenation. For control groups, the cells were cultured in a normal incubation chamber for identical periods.

### 2.4. Cell Viability Assay

Cardiomyocytes were cultured in 24-well plates and pretreated with or without gAd followed by hypoxia/reoxygenation. 3-(4,5-Dimethylthiazol-2-yl)-2,5-diphenyltetrazolium bromide (0.5 mg/ml) was added and cultured for 4 h, and then, absorbance at 490 nm was measured as described previously [21].

### 2.5. Double Labeling with Cleaved Caspase-3 Immunofluorescence and TUNEL Staining

After hypoxia/reoxygenation, the cells were washed three times with PBS at room temperature, fixed with 4% paraformaldehyde for 10 min, and stained with an anti-cleaved caspase-3 antibody at 4°C overnight. TUNEL staining was performed using an apoptosis detection kit (Roche, South San Francisco, CA, USA). Hoechst 33342 was used for nuclear counterstaining. Stained cells were imaged using a fluorescence microscope (Olympus BX51, Tokyo, Japan).

### 2.6. TUNEL Staining in Heart Section

Fresh hearts from rats were fixed in 4% paraformaldehyde, dehydrated in ethanol, and embedded in paraffin. Then, 7 *μ*m sections from the heart were obtained, deparaffinized, rehydrated, and performed antigen retrieval in 10 mM sodium citrate buffer. The cardiomyocytes of sections were detected by a TUNEL reaction mixture (In Situ Cell Death Detection Kit, POD; Roche, Mannheim, Germany) as described previously [17].

### 2.7. Immunofluorescent Staining for NF-*κ*B

Cells were washed with PBS, fixed with 4% paraformaldehyde, permeabilized with Triton X-100, blocked with bovine serum albumin, and stained with a rabbit anti-NF-*κ*B (p65) antibody (1 : 200 dilution) at 4°C overnight followed by incubation with an anti-rabbit IgG, HRP-linked antibody. Nuclear counterstaining was with Hoechst 33342. A fluorescence microscope was used to examine stained cells.

### 2.8. Lactate Dehydrogenase (LDH) Release

LDH release in culture media was measured by using an LDH assay kit (SolarBio, Beijing, China) according to the manufacturer's instructions.

### 2.9. *In Situ* Detection of Reactive Oxygen Species (ROS)

Frozen, unfixed myocardial cells were stained with 10 *μ*M dihydroethidium, a ROS probe, for 30 min in a dark humidified chamber at 37°C. ROS was imaged using a fluorescence microscope and quantified using NIH ImageJ software (https://imagej.nih.gov).

### 2.10. Measurement of Total Superoxide Dismutase (T-SOD) and Malondialdehyde (MDA)

T-SOD was measured using hydroxylamine method-based T-SOD detection kits (Jiancheng Bioengineering Institute, Nanjing, China). MDA was determined using a thiobarbituric acid detection kit (Jiancheng Bioengineering Institute). All measurements were conducted according to the manufacturer's instructions.

### 2.11. Western Blotting Assay

Protein (10-20 *μ*g) from NRVM lysate and the left ventricular tissues were separated using SDS-polyacrylamide gels (12%) and electrically transferred to polyvinylidene difluoride (PVDF) membranes (Millipore, Danvers, MA, USA). The membranes were probed with an indicated rabbit primary antibody (1 : 1000 dilution) (cleaved caspase-3, RIP1, RIP3, NF-*κ*B, p38MAPK, phosphorylated-NF-*κ*B, phosphorylated-p38MAPK, Bcl-2, Bax, or GAPDH), incubated with a goat anti-rabbit secondary antibody (1 : 3000 dilution), and visualized using ECL (Millipore). Densitometric analysis was performed using the NIH ImageJ measuring software. Average densitometry values were obtained from 3 to 5 independent experiments.

### 2.12. Myocardial I/R Model in Rat

Healthy male Sprague-Dawley (SD) rats (220–250 g, 7–8 weeks old) were anesthetized by intraperitoneal injection of pentobarbital sodium (45 mg/kg). SD rats were intubated and the heart and left anterior descending coronary artery exposed. The left anterior descending coronary artery was occluded with 5-0 silk thread to ischemia for 45 minutes, and then, the suture was released to reperfuse the heart for 3 hours. The rats were randomly divided into three groups: (1) sham group (sham)—the rats were subjected to the same surgical procedure but the sutures around the coronary arteries were not tightened (*n* = 6); (2) I/R group—rats were injected with normal saline 10 minutes before reperfusion (*N* = 6); and (3) I/R+gAd group—rats were injected with gAd (1 mg/g) within 10 minutes before reperfusion (*n* = 6). After reperfusion, the heart tissue was collected and used for subsequent experiments.

### 2.13. Statistical Analysis

All data are given as the mean and SEM (standard error of the mean) and analyzed using GraphPad Prism 6.0, GraphPad Software, San Diego, CA, USA. The difference between two groups was assessed using Student's *t*-test, and multiple comparisons were analyzed using one-way ANOVA with the Bonferroni correction. Differences between groups were considered to be significant at *P* < 0.05.

## 3. Results

### 3.1. Globular Adiponectin Attenuates Hypoxia/Reoxygenation-Induced Primary Neonatal Cardiomyocyte Injury

**Figure 1:**
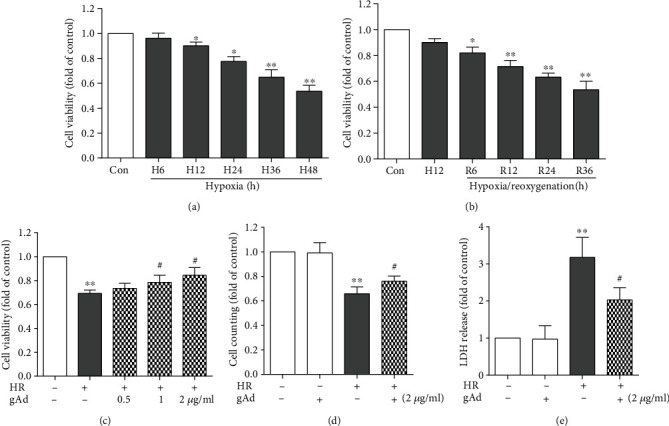
Globular adiponectin (gAd) improved cell viability during hypoxia/reoxygenation. (a) Various time points of hypoxia (0, 6, 12, 24, 36, and 48 h) reduced the NRVM viability as demonstrated by MTT assay (*n* = 5). (b) After 12 h of hypoxia and various reoxygenation periods (0, 6, 12, 24, and 36), the cell viability was significantly reduced compared with that of the control group (*n* = 5). (c) The cell viability significantly increased following pretreatment of the cardiomyocytes with various concentrations of gAd (0, 0.5, 1, and 2 *μ*g/ml) (*n* = 5). (d) The 2 *μ*g/ml dose of gAd increased the number of NRVM cells remaining after hypoxia/reoxygenation (*n* = 5). (e) The 2 *μ*g/ml dose of gAd reduced the release of LDH in the culture medium compared with the HR model group (*n* = 5). ^∗^*P* < 0.05 and ^∗∗^*P* < 0.01 vs. the control group; ^#^*P* < 0.05 and ^##^*P* < 0.01 vs. the H12/R12 group.

First, we used the MTT assay to determine cardiomyocyte viability at different time points following the exposure to hypoxia. As shown in (Figure 1(a)), a time-dependent, significant decrease in viable cells was noted, except at 6 h, as compared to the baseline level. A similar analysis was further conducted for reoxygenation (Figure 1(b)). A more remarkable decrease was seen in cardiomyocytes 6, 12, 24, and 36 h following reoxygenation as compared to cardiomyocytes exposed to hypoxia alone. A moderate injury in cardiomyocytes was observed following 12 h hypoxia/12 h reoxygenation, with approximately 30% reduction in cardiomyocyte viability. Therefore, we used 12 h hypoxia/12 h reoxygenation in all our experiments.

Next, we investigated the effect of gAd treatment on hypoxia/reoxygenation-induced cardiomyocyte injury. Pretreatment with gAd at 0.5, 1, or 2 *μ*g/ml significantly increased cardiomyocyte viability (Figure 1(c)), suggesting the protection against hypoxia/reoxygenation-induced cardiomyocyte injury. This was further confirmed in cell counting and LDH release assay, as indicated by the increased number of cardiomyocytes and reduced LDH release in culture supernatants (Figures 1(d) and 1(e)). These results indicate that gAd treatment improves hypoxia/reoxygenation-induced cardiomyocyte injury.

### 3.2. Globular Adiponectin Attenuates Hypoxia/Reoxygenation-Induced Necroptosis and Apoptosis in Cardiomyocytes

**Figure 2:**
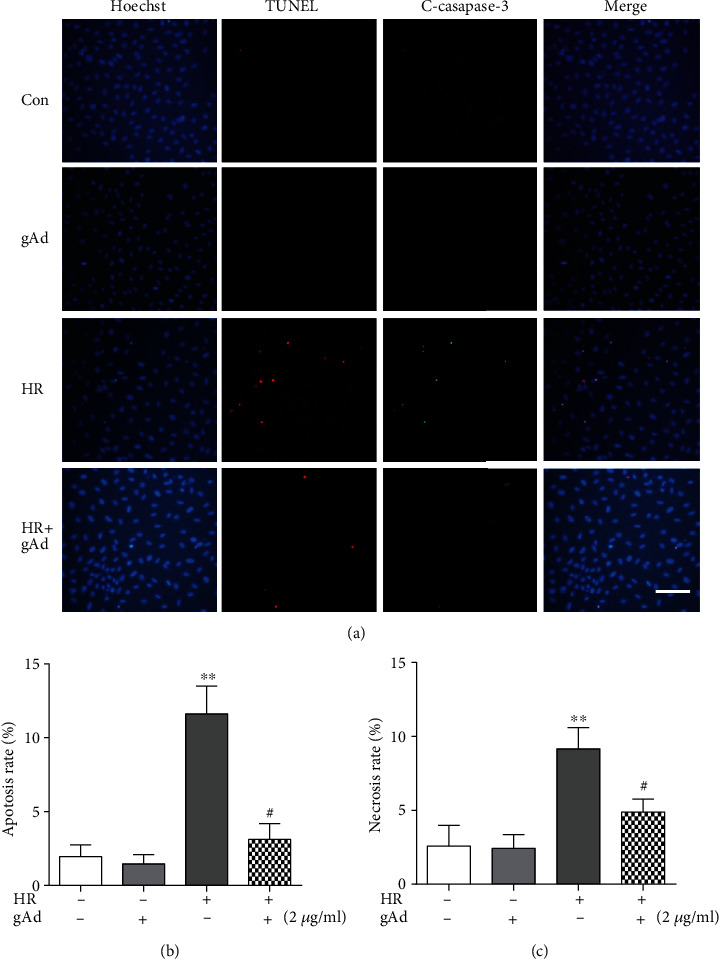
gAd protected NRVM cells against hypoxia/reoxygenation-induced cell death. (a) TUNEL (red) and cleaved caspase-3 (green) staining, in immunofluorescent images, was performed, and nuclei were stained with Hoechst 33342 (blue), which showed that apoptotic and necroptotic cells were increased after hypoxia/reoxygenation. Cells positive for both cleaved caspase-3 and TUNEL were apoptotic, whereas cleaved caspase-3-negative but TUNEL-positive cells were considered to be necroptotic (scale bar, 20 *μ*m). (b, c) The bar graph shows the average data for apoptotic and necroptotic cells (*n* = 10). ^∗∗^*P* < 0.01 vs. the control group; ^#^*P* < 0.01 vs. the H12/R12 group.

Given the importance of cell death in myocardial ischemia/reperfusion injury, to assess the effect of gAd on hypoxia/reoxygenation-induced cell death and further define the types of cell death, we costained the cells using a TUNEL assay and anti-cleaved caspase-3 antibodies, which allowed differentiating necroptotic cells (cleaved caspase-3^−^TUNEL^+^) from apoptotic cells (cleaved caspase-3^+^TUNEL^+^). As seen in Figure 2(a), hypoxia/reoxygenation significantly increased the proportions of necroptosis and apoptosis in cardiomyocytes as compared to control cells. In contrast, pretreatment with gAd led to a 75% and 50% reduction in hypoxia/reoxygenation-induced necroptosis and apoptosis, respectively, in cardiomyocytes (Figures 2(b) and 2(c)). Thus, these data indicate that, in addition to apoptosis, hypoxia/reoxygenation induces necroptosis and was protected by gAd treatment.

### 3.3. Globular Adiponectin Attenuated Hypoxia/Reoxygenation-Induced Cardiomyocyte Injury in Part by Inhibiting Necroptosis

**Figure 3:**
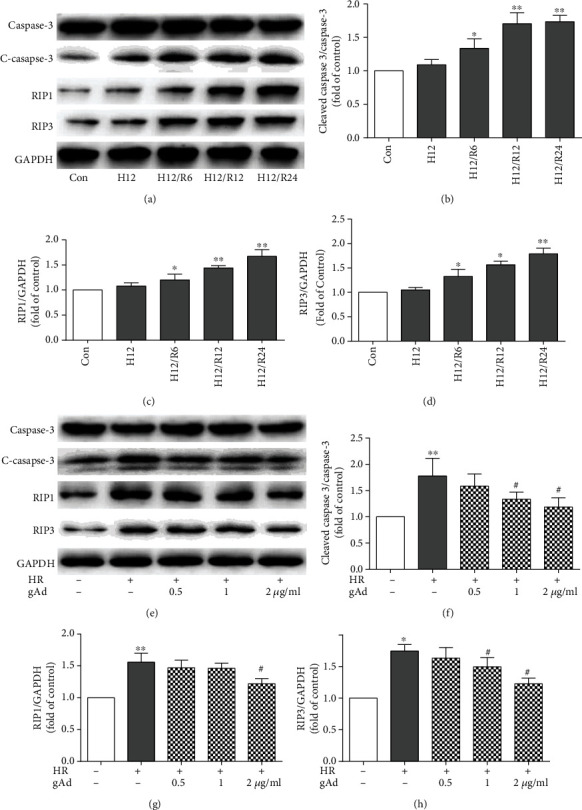
gAd could attenuate hypoxia/reoxygenation-induced cell apoptosis. (a) Hypoxia/reoxygenation (H12) and R0, 6, 12, and 24 h response of activated (cleaved) caspase-3, RIP1, and RIP3 as assayed by Western blotting for NRVMs (*n* = 3). (e) gAd (0, 0.5, 1, and 2 *μ*g/ml) suppressed hypoxia/reoxygenation-induced (cleaved) caspase-3, RIP1, and RIP3 as activation in a dose-dependent manner (*n* = 4). (b–d, f–h) The bar figures show the protein expression rates of (cleaved) caspase-3, RIP1, and RIP3, respectively.^∗^*P* < 0.05 and ^∗∗^*P* < 0.01 vs. the control group; ^#^*P* < 0.05 vs. the H12/R12 group.

Cleaved caspase-3 is widely accepted as a specific marker for apoptosis, while RIP3 and RIP1 are used as molecular markers for necroptosis [9]. Thus, we used these markers to further define the types of cell death following hypoxia/reoxygenation. As shown in Western blot analysis, hypoxia/reoxygenation induced a time-dependent increase in the protein levels of cleaved caspase-3, RIP3, and RIP1 (Figures 3(a)–3(d)). Similar to TUNEL and cleaved caspase-3 immunofluorescence staining, necroptosis and apoptosis were induced simultaneously in cardiomyocytes following hypoxia/reoxygenation.

Next, we investigated whether gAd influences hypoxia/reoxygenation-induced necroptosis as well as apoptosis in cardiomyocytes. As shown in Western blot analysis (Figures 3(e)–3(h)), gAd treatment markedly reduced the expression levels of cleaved caspase-3, RIP1, and RIP3 proteins in a dose-dependent manner. These data further strengthen immunofluorescence findings that gAd inhibited both necroptosis and apoptosis in cardiomyocytes following hypoxia/reoxygenation.

### 3.4. Pharmacological Inhibition of Caspases or RIP1 Simulates the Suppression of Hypoxia/Reoxygenation-Induced Cardiomyocyte Necroptosis and Apoptosis by gAd

**Figure 4:**
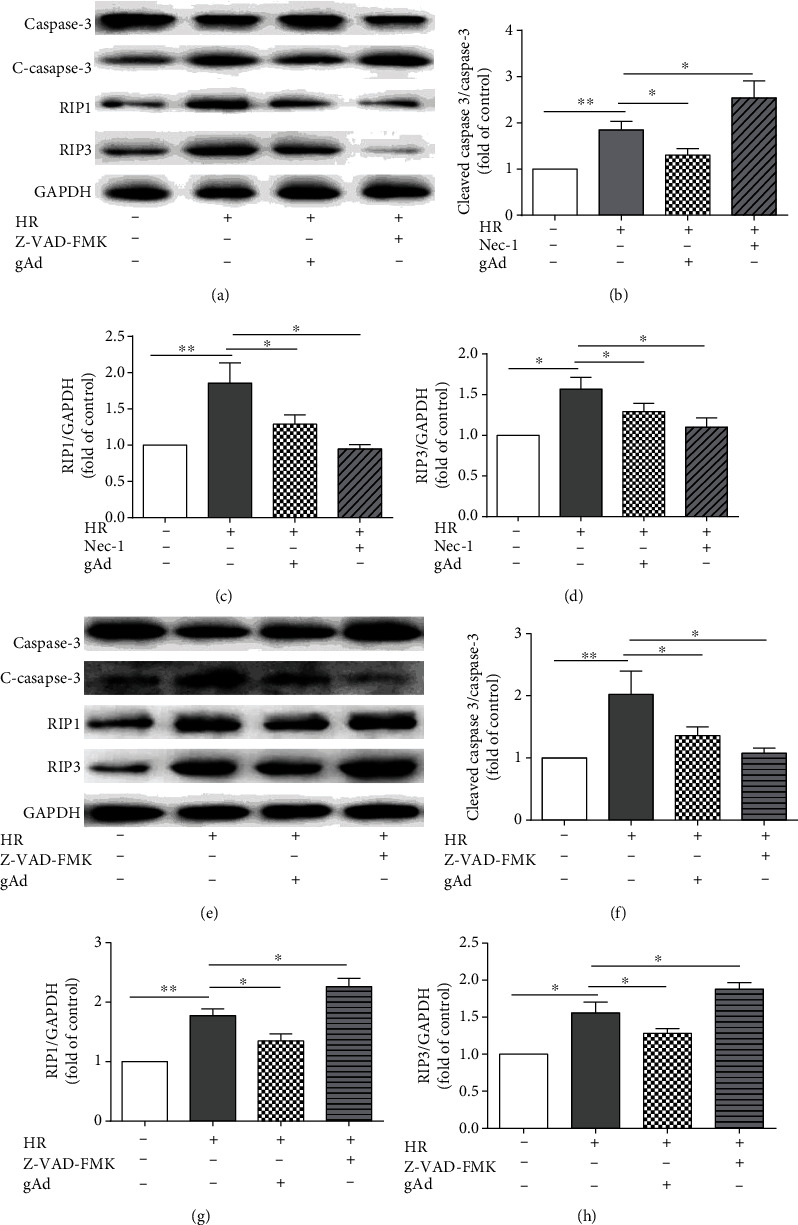
The inhibition efficiency of gAd in hypoxia/reoxygenation-induced apoptosis and necroptosis compared with nec-1 and Z-VAD-FMK. (a, c, d) Western blotting shows that necrostatin-1 (nec-1, 100 mM), a specific blocker of necroptosis, inhibited the level of RIP1 and RIP3 and increased the level of cleaved caspase-3 (*n* = 4). (b) The expression of cleaved caspase-3 was increased in the nec-1 group after H/R (*n* = 4). (e, f) Z-VAD-FMK (50 mM), a cell-permeable pan-caspase inhibitor, suppresses the cleaved caspase-3 and increased the RIP1 and RIP3 expression (*n* = 4). (g, h) The expressions of RIP1 and RIP3were increased in the Z-VAD-FMK group after H/R (*n* = 4). ^∗^*P* < 0.05; ^∗∗^*P* < 0.01.

Because gAd attenuated hypoxia/reoxygenation-augmented expression levels of RIP1 and RIP3, key signaling molecules for necroptosis, in cardiomyocytes, we evaluated whether treatment of cardiomyocytes with necrostatin-1 (nec-1), a specific inhibitor to RIP1, mimics gAd-mediated suppression of necroptosis in cardiomyocytes. Treatment with necrostatin-1 reduced the expression levels of RIP1 and RIP3, while increasing the expression levels of cleaved caspase-3, in cardiomyocytes after hypoxia/reoxygenation (Figures 4(a)–4(d)). On the other hand, treatment with Z-VAD-FMK, a pan-caspase inhibitor, reduced cleaved caspase-3 levels (Figures 4(e) and 4(f)), while increasing the expression levels of RIP1 and RIP3 (Figures 4(e), 4(g), and 4(h)). These results demonstrated that, unlike necrostatin-1 and Z-VAD-FMK, gAd target both apoptosis and necroptosis during hypoxia/reoxygenation.

### 3.5. Globular Adiponectin Treatment Reduces Hypoxia/Reoxygenation-Induced Oxidative Stress

**Figure 5:**
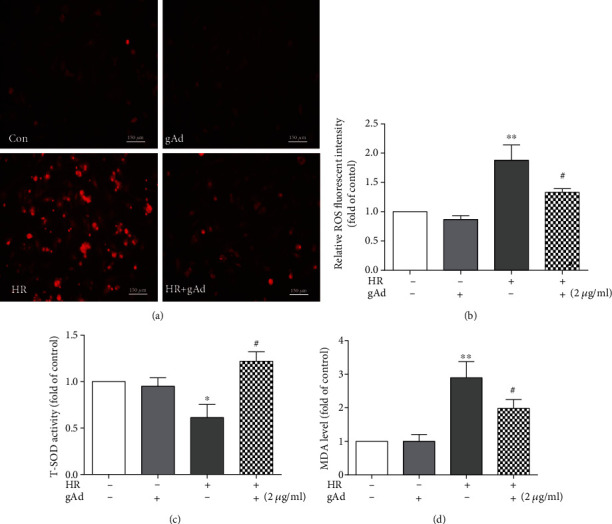
gAd protected cardiomyocyte by attenuation of oxidative stress and activation of the p38/NF-*κ*B signaling pathway during hypoxia/reoxygenation. (a) The ROS levels were measured using DHE (red) staining (*n* = 4, scale bar, 100 *μ*m). (b) The bar figures show the ROS fluorescent intensity. (c) T-SOD activities were examined using the hydroxylamine method (*n* = 7). (d) The MDA levels were examined using the TBA method (*n* = 4).

Because of the importance of oxidative stress in necroptosis and apoptosis, we examined whether gAd treatment alters ROS generation, antioxidant enzymes, and consequently oxidative lipid damage during hypoxia/reoxygenation. As shown in (Figures 5(a) and 5(b)), gAd treatment substantially decreased the ROS levels, as measured via a fluorescent superoxide probe, in cardiomyocytes following hypoxia/reoxygenation as compared to vehicle treatment. The suppression of total superoxide dismutase (T-SOD) by hypoxia/reoxygenation was reversed, even augmented, following gAd treatment (Figure 5(c)). Consequently, oxidative lipid damage, MDA, induced by hypoxia/reoxygenation, was attenuated by gAd treatment (Figure 5(d)). Thus, these results suggest that the suppression of necroptosis and apoptosis by gAd may be in part mediated by its antioxidative activity.

### 3.6. Globular Adiponectin Inhibits Hypoxia/Reoxygenation-Induced MAPK/NF-*κ*B Signaling and Promotes Antiapoptotic Bcl-2 Expression

**Figure 6:**
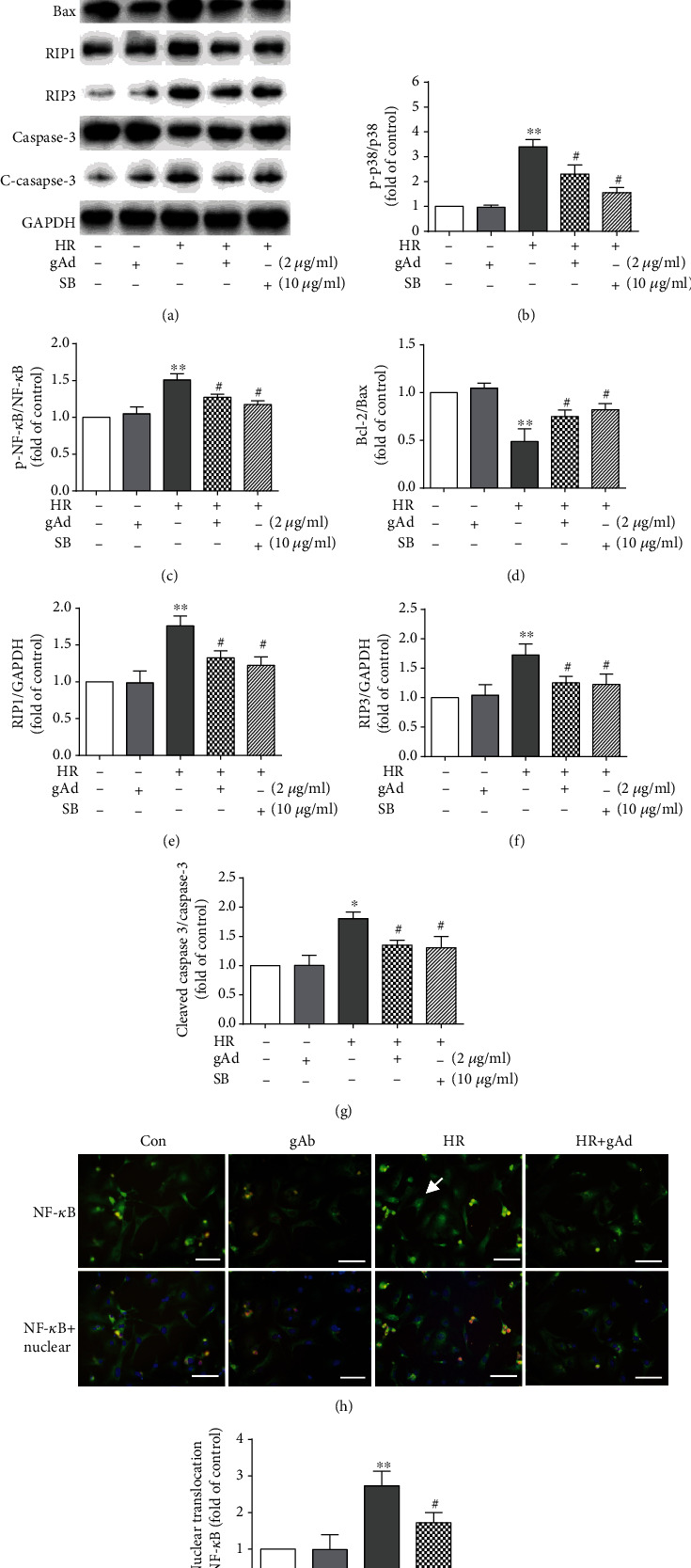
gAd inhibits hypoxia/reoxygenation-induced necroptosis and apoptosis in cardiomyocytes by disrupting the p38/NF-*κ*B signaling pathway. (a–g) Western blotting and average data of p38, NF-*κ*B, Bcl-2, Bax, RIP1, RIP3, and (cleaved) caspase-3 protein abundance in the control, gAd, HR, HR+gAd, and HR+SB groups (*n* = 3). (h) Immunofluorescence double staining for NF-*κ*B (green) localization. The nuclei of the cells were counterstained with Hoechst (blue) (*n* = 3, scale bar, 50 *μ*m). (i) The bar figures show the quantification data on NF-*κ*B translocation. ^∗^*P* < 0.05 and ^∗∗^*P* < 0.01 vs. the control group; ^#^*P* < 0.05 vs. the H/R group.

To further investigate the molecular mechanism by which gAd protected hypoxia/reoxygenation-treated cardiomyocyte necroptosis and apoptosis, using Western blotting assay, we examined whether gAd treatment alters MAPK, NF-*κ*B, proapoptotic Bax, and antiapoptotic Bcl-2 signaling pathways, all important for necroptosis and apoptosis. As shown in (Figure 6), hypoxia/reoxygenation enhanced the activity of MAPK and NF-*κ*B as indicated by increased levels of p-p38 (Figure 6(b)) and p-NK-*κ*B (Figure 6(c)), while leading to a relatively increased proapoptotic activity as assayed by a reduced ratio of Bcl-2/Bax (Figure 6(d)), with increased expression of RIP1 (Figure 6(e)), RIP3 (Figure 6(f)), and cleaved caspase-3 (Figure 6(g)). Additionally, immunofluorescence staining confirmed nuclear translocation of NF-*κ*B (Figures 6(h) and 6(i)). In contrast, all these influences were largely reversed by gAd treatment. Furthermore, SB203580, the inhibitor for p38, was used, and its effect on hypoxia/reoxygenation-induced cardiomyocyte injury was compared with that of globular adiponectin. The results show that compared with globular adiponectin, SB203580 can also inhibit p38 phosphorylation during hypoxia/reoxygenation. In addition, inhibitions of the p38MAPK pathways can partially inhibit the hypoxia/reoxygenation-induced Bcl-2/Bax ratio and decrease the expression of RIP1, RIP3, and cleaved caspase-3 in cardiomyocyte injury. These results suggest that gAd may protect necroptosis and apoptosis in cardiomyocytes by attenuating p38MAPK/NF-*κ*B signaling and by favoring antiapoptotic Bcl-2 expression.

### 3.7. gAd Inhibits Both Apoptosis and Necroptosis in the Myocardial Ischemia/Reperfusion Injury *In Vivo*

**Figure 7:**
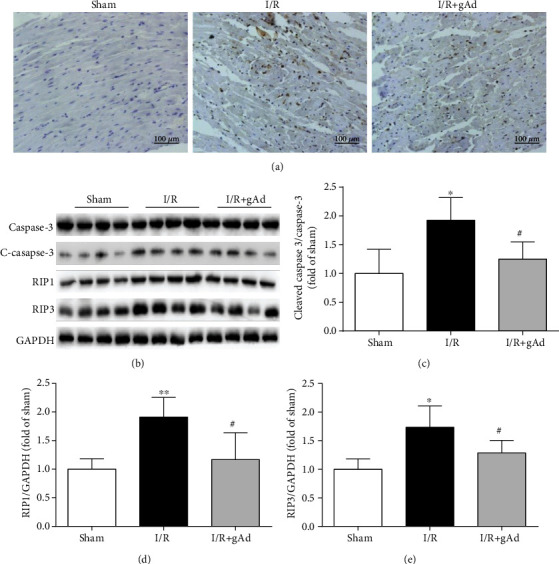
gAd inhibits both apoptosis and necroptosis in the myocardial ischemia/reperfusion rat model. (a) Representative images of the TUNEL staining. (b–e) Western blotting and average data of RIP1, RIP3, and (cleaved) caspase-3 protein abundance in the sham, I/R, and I/R+gAd groups (*n* = 6). ^∗^*P* < 0.05 and ^∗∗^*P* < 0.01 vs. the sham group; ^#^*P* < 0.05 vs. the I/R group.

We further analyze gAd's function on both apoptosis and necroptosis in a myocardial ischemia/reperfusion (I/R) model in rats. TUNEL staining proved that compared with the sham group, the rate of TUNEL-positive cells is significantly higher in the I/R group. And gAd could decrease the TUNEL-positive cell rate in the I/R+gAd group (Figure 7(a)). Additionally, gAd could also suppress the expression levels of cleaved caspase-3, RIP1, and RIP3 after I/R treatment, which confirms that gAd inhibits both apoptosis and necroptosis in the myocardial ischemia/reperfusion injury in vivo (Figures 7(b)–7(e)).

## 4. Discussion

Polypeptide globular adiponectin is an adipocyte-derived cytokine that reduces the risk for acute coronary syndromes [17, 18]. We and others have previously reported that exogenous gAd treatment alleviated hypoxia/reoxygenation-induced injury in cardiomyocytes [15, 17, 18]. However, the underlying mechanisms for gAd-mediated cardioprotection remain unclear. For many years, apoptosis has been considered to be the sole form of regulated cell death and has also been the focus in myocardial ischemia/reperfusion-induced injury [9, 11]. Necroptosis is a novel discovered programmed cell death, shares some common features with necrosis, and is involved in cardiovascular diseases [8, 9]. Thus, the present study investigated whether gAd could protect cardiomyocytes by regulating necroptosis in a cardiomyocyte hypoxia/reoxygenation model and in a myocardial ischemia/reperfusion (I/R) rat model.

Cell death occurs via programmed cell death (apoptosis), regulated necrosis (necroptosis), and/or nonregulated necrosis [11]. In this study, we found that both apoptosis and necrosis increased simultaneously following hypoxia/reoxygenation. In Western blot analysis, as expected, hypoxia/reoxygenation induced a time-dependent increase in the protein levels of cleaved caspase-3, a marker for apoptosis in cardiomyocytes [9]. Interestingly, hypoxia/reoxygenation also substantially increased the levels of RIP3 and RIP1, which form the necrosome and results in necroptosis. Thus, our study demonstrates that, in addition to apoptosis, hypoxia/reoxygenation induces necroptosis in cardiomyocytes.

Consistent with previous studies, our study confirmed that gAd reduced cell death and promoted cell survival in cardiomyocytes following hypoxia/reoxygenation. More importantly, we demonstrated mechanistically that, in addition to its antiapoptotic property, the attenuation of hypoxia/reoxygenation-induced cardiomyocyte death was in part mediated by inhibiting necroptosis. Many animal experiments have demonstrated the protection of exogenous adiponectin against cardiovascular damage following ischemia/reperfusion [22, 23]. In published studies, necrostatin-1, a specific necroptosis inhibitor, inhibited necroptosis while promoting apoptotic cell death [24–26]. Conversely, Z-VAD-FMK, a pan-caspase apoptosis inhibitor, inhibited apoptosis while augmenting necroptosis [27]. In agreement with the functionally reciprocal role of apoptosis and necroptosis in cellular demise as previously proposed [25, 26], we showed the reciprocal influence on the expression of apoptotic and necroptotic proteins when cardiomyocytes were treated with necrostatin-1 or Z-VAD-FMK. Distinct from either Z-VAD-FML (inhibit apoptosis) or necrostatin-1 (inhibit necroptosis), gAd treatment resulted in simultaneous inhibition of necroptosis and apoptosis. Thus, gAd may be a more effective and ideal drug for treating ischemia-reperfusion injury.

It is well known that increased reactive oxygen species (ROS) induces cell death via structural and functional alterations in cardiomyocyte ischemia/reperfusion [9]. Apoptosis and necroptosis have been reported in reperfusion injury, in association with enhanced ROS [9]. Further, consistent with published studies [28, 29], the present study showed that pretreatment with gAd increased SOD levels, reduced ROS production, and attenuated oxidative lipid damage following cardiomyocyte hypoxia/reoxygenation. Thus, our results imply that the protection of hypoxia/reoxygenation-induced cardiomyocyte necroptosis and apoptosis by gAd may be in part mediated by its antioxidative activity.

MAPK and NF-*κ*B are involved in many biological processes [2, 3]. MK2 is an effector kinase, the downstream of MAPK and NF-*κ*B, directly phosphorylates RIP1 at residue S321, curtails RIP1 integration into cytoplasmic cytotoxic complexes, and thus suppresses RIP1-kinase-dependent apoptosis and necroptosis [30–32]. In ischemia-reperfusion injury, NF-*κ*B activators phosphorylate the I*κ*B protein, leading to rapid degradation of the I*κ*B protein through the ubiquitin-proteasome pathway and NF-*κ*B nuclear translocation [33]. RIP3 overexpression also activates the NF-*κ*B signal pathway [34, 35]. Published studies have shown the contribution of p38MAPK/NF-*κ*B signaling to necroptosis in cardiomyocytes and other cells [36–38]. In the present study, we found increased levels of p-p38MAPK and p-NF-*κ*B parallel to the occurrence of apoptosis and necroptosis. Further, gAd treatment was able to inhibit p-p38 and p-p65 as well as necroptosis and apoptosis. Additionally, gAd treatment inhibited hypoxia/reoxygenation-induced NF-*κ*B nuclear translocation. These results suggest that gAd treatment protected against hypoxia/reoxygenation-induced necroptosis and apoptosis in cardiomyocytes by disrupting p38MAPK and NF-*κ*B signaling pathways.

Our study has some limitations. First, gAd inhibited hypoxia/reoxygenation-induced necroptosis and apoptosis as effectively as necrostatin-1 or Z-VAD-FMK did; thus, we did not examine whether gAd additively or synergistically inhibited cardiomyocyte necroptosis or apoptosis when cotreated with necrostatin-1 or Z-VAD-FMK. Additionally, necroptosis and ROS generation induced by hypoxia/reoxygenation in cardiomyocytes were almost completely abrogated by gAd treatment. It is thus difficult to directly test whether antioxidative activity of gAd mediated cardiomyocyte protection against hypoxia/reoxygenation using any inhibitors to antioxidant components.

## 5. Conclusion

In conclusion, we demonstrated that globular adiponectin inhibited hypoxia/reoxygenation-induced necroptosis and apoptosis in cardiomyocytes by attenuating oxidative stress and p38MAPK/NF-*κ*B signaling pathways. Dual inhibition of necroptosis and apoptosis suggests that globular adiponectin may be more effective in treating ischemia/reperfusion-induced cardiomyocyte injury than reagents targeting apoptosis or necroptosis alone.

## Data Availability

The datasets used and/or analyzed during the current study are available from the corresponding authors on reasonable request.

## References

[1] Pan Y., Qian J. X., Lu S. Q. (2017). Protective effects of tanshinone IIA sodium sulfonate on ischemia-reperfusion-induced myocardial injury in rats. *Iranian Journal of Basic Medical Sciences*.

[2] Guo X., Jiang H., Yang J. (2016). Radioprotective 105 kDa protein attenuates ischemia/reperfusion-induced myocardial apoptosis and autophagy by inhibiting the activation of the TLR4/NF-*κ*B signaling pathway in rats. *International Journal of Molecular Medicine*.

[3] Hernández G., Lal H., Fidalgo M. (2011). A novel cardioprotective p38-MAPK/mTOR pathway. *Experimental Cell Research*.

[4] Zheng Y., Gu S., Li X. (2017). Berbamine postconditioning protects the heart from ischemia/reperfusion injury through modulation of autophagy. *Cell Death Dis*.

[5] Zhang T., Zhang Y., Cui M. (2016). CaMKII is a RIP3 substrate mediating ischemia- and oxidative stress-induced myocardial necroptosis. *Nature Medicine*.

[6] Murphy E., Steenbergen C. (2008). Mechanisms underlying acute protection from cardiac ischemia-reperfusion injury. *Physiological Reviews*.

[7] du L., Shen T., Liu B. (2017). Shock wave therapy promotes cardiomyocyte autophagy and survival during hypoxia. *Cellular Physiology and Biochemistry*.

[8] Adameova A., Goncalvesova E., Szobi A., Dhalla N. S. (2016). Necroptotic cell death in failing heart: relevance and proposed mechanisms. *Heart Failure Reviews*.

[9] Gao X., Zhang H., Zhuang W. (2015). PEDF and PEDF-derived peptide 44mer protect cardiomyocytes against hypoxia-induced apoptosis and necroptosis via anti-oxidative effect. *Scientific Reports*.

[10] Chen W., Wu J., Li L. (2015). Ppm1b negatively regulates necroptosis through dephosphorylating Rip3. *Nature Cell Biology*.

[11] Galluzzi L., Vitale I., Aaronson S. A. (2018). Molecular mechanisms of cell death: recommendations of the Nomenclature Committee on Cell Death 2018. *Cell Death and Differentiation*.

[12] Tang D., Kang R., Berghe T. V., Vandenabeele P., Kroemer G. (2019). The molecular machinery of regulated cell death. *Cell Research*.

[13] Zhe-Wei S., Li-Sha G., Yue-Chun L. (2018). The role of necroptosis in cardiovascular disease. *Frontiers in Pharmacology*.

[14] Palanivel R., Fang X., Park M. (2007). Globular and full-length forms of adiponectin mediate specific changes in glucose and fatty acid uptake and metabolism in cardiomyocytes. *Cardiovascular Research*.

[15] Han X., Wu Y., Liu X. (2017). Adiponectin improves coronary no-reflow injury by protecting the endothelium in rats with type 2 diabetes mellitus. *Bioscience Reports*.

[16] Onay-Besikci A., Altarejos J. Y., Lopaschuk G. D. (2004). gAd-globular head domain of adiponectin increases fatty acid oxidation in newborn rabbit hearts∗. *The Journal of Biological Chemistry*.

[17] Guo J., Bian Y., Bai R., Li H., Fu M., Xiao C. (2013). Globular adiponectin attenuates myocardial ischemia/reperfusion injury by upregulating endoplasmic reticulum Ca^2+^-ATPase activity and inhibiting endoplasmic reticulum stress. *Journal of Cardiovascular Pharmacology*.

[18] Bian Y. F., Hao X. Y., Gao F., Yang H. Y., Zang N., Xiao C. S. (2011). Adiponectin attenuates hypoxia/reoxygenation-induced cardiomyocyte injury through inhibition of endoplasmic reticulum stress. *Journal of Investigative Medicine*.

[19] Lu Y., Bian Y., Wang Y., Bai R., Wang J., Xiao C. (2015). Globular adiponectin reduces vascular calcification via inhibition of ER-stress-mediated smooth muscle cell apoptosis. *International Journal of Clinical and Experimental Pathology*.

[20] Shen T., Aneas I., Sakabe N. (2011). Tbx 20 regulates a genetic program essential to adult mouse cardiomyocyte function. *The Journal of Clinical Investigation*.

[21] Cao Y., Shen T., Huang X. (2017). Astragalus polysaccharide restores autophagic flux and improves cardiomyocyte function in doxorubicin-induced cardiotoxicity. *Oncotarget*.

[22] Shibata R., Sato K., Pimentel D. R. (2005). Adiponectin protects against myocardial ischemia-reperfusion injury through AMPK- and COX-2-dependent mechanisms. *Nature Medicine*.

[23] Potenza M. A., Sgarra L., Nacci C., Leo V., De Salvia M. A., Montagnani M. (2019). Activation of AMPK/SIRT1 axis is required for adiponectin-mediated preconditioning on myocardial ischemia-reperfusion (I/R) injury in rats. *PLoS One*.

[24] Liu J., Wu P., Wang Y. (2016). Ad-HGF improves the cardiac remodeling of rat following myocardial infarction by upregulating autophagy and necroptosis and inhibiting apoptosis. *American Journal of Translational Research*.

[25] Northington F. J., Chavez-Valdez R., Graham E. M., Razdan S., Gauda E. B., Martin L. J. (2011). Necrostatin decreases oxidative damage, inflammation, and injury after neonatal HI. *Journal of Cerebral Blood Flow and Metabolism*.

[26] Han W., Xie J., Fang Y., Wang Z., Pan H. (2012). Nec-1 enhances shikonin-induced apoptosis in leukemia cells by inhibition of RIP-1 and ERK1/2. *International Journal of Molecular Sciences*.

[27] Polito L., Bortolotti M., Pedrazzi M., Mercatelli D., Battelli M. G., Bolognesi A. (2016). Apoptosis and necroptosis induced by stenodactylin in neuroblastoma cells can be completely prevented through caspase inhibition plus catalase or necrostatin-1. *Phytomedicine*.

[28] Essick E. E., Ouchi N., Wilson R. M. (2011). Adiponectin mediates cardioprotection in oxidative stress-induced cardiac myocyte remodeling. *American Journal of Physiology. Heart and Circulatory Physiology*.

[29] Tao L., Gao E., Jiao X. (2007). Adiponectin cardioprotection after myocardial ischemia/reperfusion involves the reduction of oxidative/nitrative stress. *Circulation*.

[30] Wu M. Y., Yiang G. T., Liao W. T. (2018). Current mechanistic concepts in ischemia and reperfusion injury. *Cellular Physiology and Biochemistry*.

[31] Jaco I., Annibaldi A., Lalaoui N. (2017). MK2 phosphorylates RIPK1 to prevent TNF-induced cell death. *Mol Cell*.

[32] Menon M. B., Gropengießer J., Fischer J. (2017). p38^MAPK^/MK2-dependent phosphorylation controls cytotoxic RIPK1 signalling in inflammation and infection. *Nature Cell Biology*.

[33] Chen Z. J., Parent L., Maniatis T. (1996). Site-specific phosphorylation of I*κ*B*α* by a novel ubiquitination-dependent protein kinase activity. *Cell*.

[34] Kasof G. M., Prosser J. C., Liu D., Lorenzi M. V., Gomes B. C. (2000). The RIP-like kinase, RIP3, induces apoptosis and NF-*κ*B nuclear translocation and localizes to mitochondria. *FEBS Letters*.

[35] Lu C., Zhou L. Y., Xu H. J. (2014). RIP3 overexpression sensitizes human breast cancer cells to parthenolide in vitro via intracellular ROS accumulation. *Acta Pharmacologica Sinica*.

[36] Qin S., Yang C., Huang W. (2018). Sulforaphane attenuates microglia-mediated neuronal necroptosis through down- regulation of MAPK/NF-*κ*B signaling pathways in LPS-activated BV-2 microglia. *Pharmacological Research*.

[37] Mármol I., Virumbrales-Muñoz M., Quero J. (2017). Alkynyl gold(I) complex triggers necroptosis via ROS generation in colorectal carcinoma cells. *Journal of Inorganic Biochemistry*.

[38] Feng T., Chen W., Zhang C. (2017). The p 38/CYLD pathway is involved in necroptosis induced by oxygen-glucose deprivation combined with ZVAD in primary cortical neurons. *Neurochemical Research*.

